# Aquafilling Filler for Buttock Augmentation Cause Severe Long-Term Complications: A Case Report

**DOI:** 10.1055/s-0045-1801866

**Published:** 2025-01-17

**Authors:** Camilla Soendergaard Kristiansen, Christian Lyngsaa Lang, Ann Haerskjold, Claus Zachariae, Anders Klit

**Affiliations:** 1 Department of Plastic Surgery and Burns Treatment, Rigshospitalet, Copenhagen, Denmark; 2 Department of Dermatology, Bispebjerg Hospital, Copenhagen, Denmark; 3 Department of Dermatology, Gentofte Hospital, Gentofte, Denmark; 4 Department of Clinical Medicine, Faculty of Health and Medical Sciences, University of Copenhagen, Copenhagen, Denmark

**Keywords:** filler injections, Aquafilling, complications

## Abstract

Filler injections for buttock augmentation are becoming more popular as a cosmetic procedure, which leads to an expected increase in the number of complications emphasizing the importance of reporting cases with both well-known, severe, and previously undiscovered complications, as well as their possible treatment strategies.

We present an 18-year-old woman who suffered severe long-term complications following Aquafilling injections for buttock augmentation, including filler migration, infection leading to septic shock, and nonparathyroid hypercalcemia, which has the potential to cause renal insufficiency. To date, we have not found any reports describing the association between nonparathyroid hypercalcemia and Aquafilling. Additionally, we outline a treatment regimen involving a minimally invasive approach, which includes daily irrigation, manual compression, and passive evacuation. At follow-up, the patient returned to her everyday life with no lasting sequelae, except for a solid mass medially on the right thigh.

## Introduction

Filler injections for buttock augmentation are becoming increasingly popular, but the outcomes and complications of newer fillers remain unclear. 1 2

Aquafilling, produced by BIOTRH, spol, s.r.o., Czech Republic, consists of 98% sodium chloride solution and 2% copolyamide. It is described as well-tolerated, biocompatible with human tissue, and yielding long-term clinical results, but recent reports indicate several complications. 2 3 4 5 6

This case report presents an 18-year-old woman who suffered severe, long-term complications after Aquafilling injections for buttock augmentation and outlines a minimally invasive treatment approach.

## Case Report

**Fig. 1:**
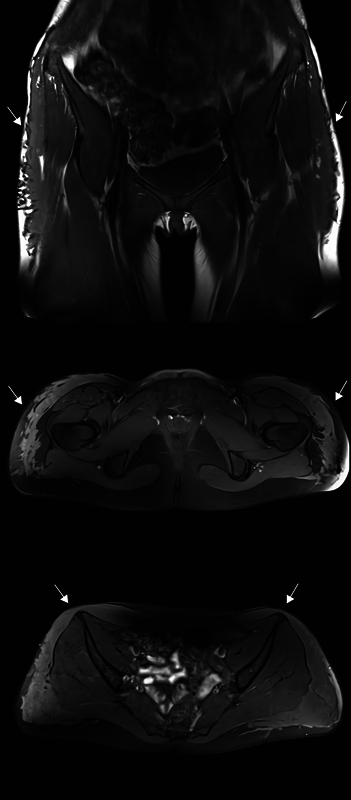
Magnetic resonance imaging (MRI) showing migration of the filler to the proximal part of both thighs and inguinal regions highlighted by arrows.

An 18-year-old woman underwent buttock augmentation with Aquafilling in Istanbul. Three years later, the patient was referred to the Department of Plastic Surgery and Burns Treatment (DPSBT), Rigshospitalet, in Copenhagen, due to increasing soreness and firmness in the anterolateral part of both thighs and inguinal regions. Magnetic resonance imaging revealed migration of the filler into these areas ( Fig. 1 ). As there were no signs of infection, no further treatment was provided.

**Fig. 2:**
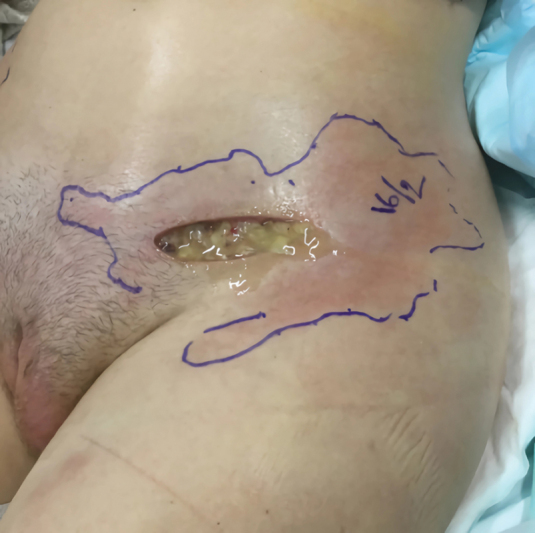
Cloudy fluid and grainy filler material from the surgical wound in the left inguinal region.

Six months later, the patient developed an abscess in the left inguinal region and diffuse skin infection in the lower abdomen. Surgery was performed with incision in the left inguinal region, revision of the infected area, and drainage of infected grainy filler material ( Fig. 2 ).

**Fig. 3:**
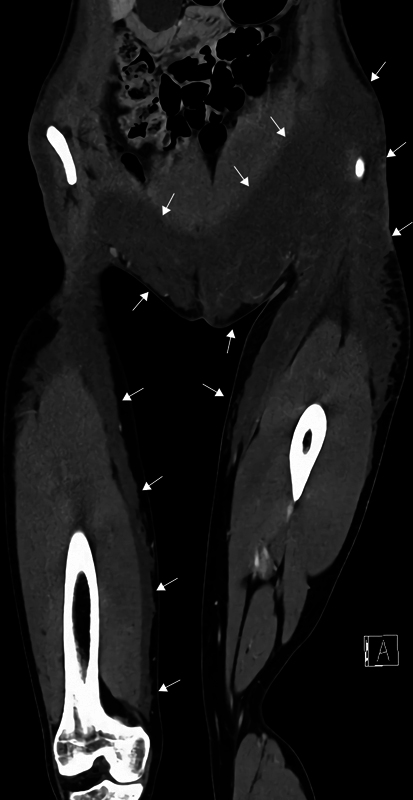
Contrast-enhanced computed tomography (CT) scan (coronal view on the level of spina iliaca anterior superior) showing migration of the filler, highlighted by arrows, extending from the hips to the lower abdomen, into both inguinal regions and the left labia majora, and down to the anteromedial part of the thighs. On the right side, the filler extends down to the level of the knee.

Despite undergoing surgery and receiving triple-therapy antibiotics (benzylpenicillin, cloxacillin, and metronidazole), the patient developed septic shock. Surgical exploration of the left inguinal and lateral gluteal region ruled out necrotizing soft tissue infection, and both incisions were left open to heal by secondary intention. Contrast-enhanced computed tomography scan revealed migration of the filler extending from both hips down to the anteromedial part of the thighs. On the right side, the filler extended to the level of the knee ( Fig. 3 ).

Following treatment of septic shock at the local intensive care unit, the patient was referred to DPSBT for further treatment.

As the infected filler material was found to be a coherent, grainy gel-like substance, making it unsuitable for aspiration, evacuation with liposuction equipment, or treatment with negative wound pressure therapy, we decided to remove the substance by irrigation, manual compression, and passive evacuation through the incisions.

The surgical wounds were irrigated several times daily with a catheter connected to a syringe. In addition, substantial amounts of filler material exited the cavities through manual compression and patient mobilization. During the admission, we achieved a gradual reduction of the filler material.

Multiple blood and wound cultures were examined. Only the wound cultures showed growth of *Staphylococcus aureus* with antimicrobial resistance to penicillin. In cooperation with specialists in biofilm formation from the Department of Microbiology, Copenhagen, and the Department of Dermatology, Gentofte Hospital, Gentofte, a treatment plan was established, and the patient received 2 weeks of intravenous vancomycin, metronidazole, clindamycin, and meropenem, leading to decreased infection markers and clinical improvement.

The patient developed nonparathyroid hypercalcemia with a gradual and concerning rise in ionized calcium levels; a condition that could potentially lead to renal insufficiency due to calcium deposition. The patient, who was young and otherwise healthy with no renal diseases, had no clinical symptoms of hypercalcemia and was treated with forced hydration through oral intake supplemented with intravenous fluids. The ionized calcium leveled off at 1.41 mmol/L and a gradual normalization of the calcium levels was achieved.

The patient was discharged with open surgical wounds to allow passive filler drainage and prescribed oral moxifloxacin to prevent biofilm formation. The patient was instructed to flush the cavities twice daily and received daily visits from a home care nurse for wound care.

**Fig. 4:**
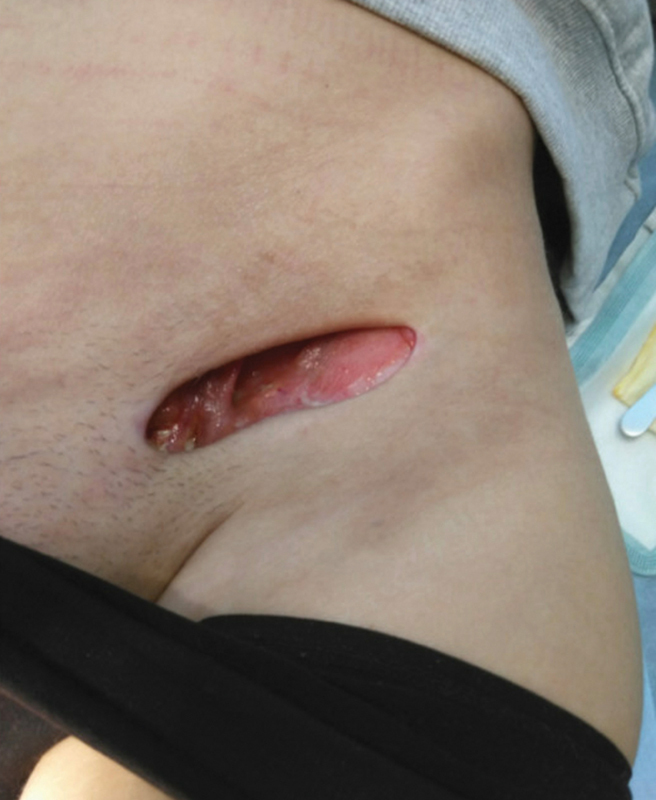
Nine weeks after discharge, postoperative appearance of the left inguinal region before the cavity was sutured.

Six weeks after discharge, the surgical wound in the left lateral gluteal region had healed by secondary intention. Nine weeks after discharge, the amount of filler material leaving the cavity in the left inguinal region had ceased. The cavity was surgically revised and sutured with no postoperative complications ( Fig. 4 ).

Three months later, the patient had similar symptoms in the right inguinal region and mons pubis. Blood samples showed elevated infection markers but normal calcium levels. The patient was treated similarly to the left side and discharged with oral antibiotics based on wound cultures showing growth of commensal microbes *Staphylococcus hominis* , *Cutibacterium acnes* , and *Peptoniphilus harei* with antimicrobial resistance for tetracycline, along with cavity flushing instructions. Eight weeks later, the right cavity was surgically revised and sutured with no complications.

**Fig. 5:**
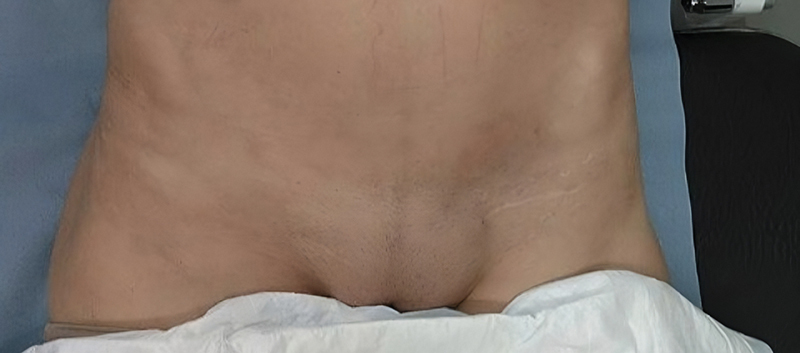
At the 24-month follow-up, there were no visible changes in the inguinal regions.

**Fig. 6:**
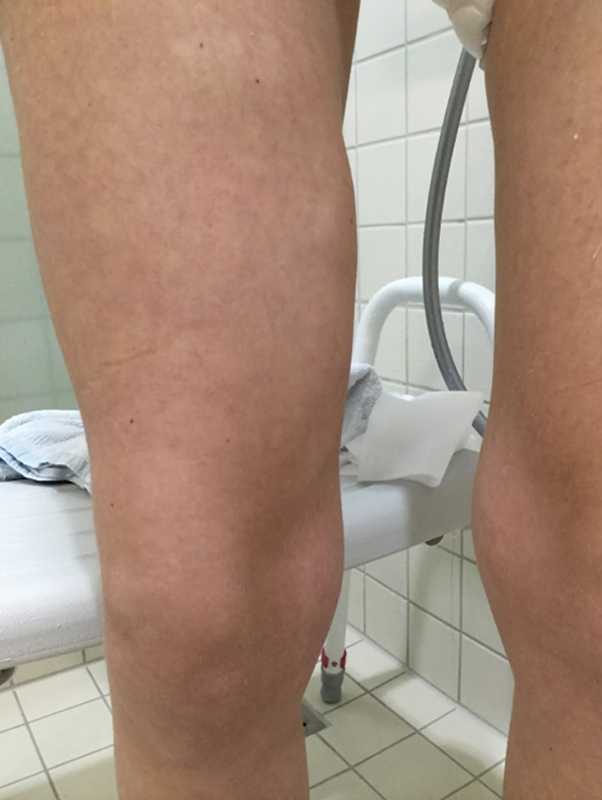
At the 24-month follow-up, the only visible change was a solid mass located medially on the right thigh.

At 3-, 6-, 13-, 18-, and 24-month follow-up, the patient had returned to her everyday life with no further infections related to the filler or lasting sequelae, except for a solid mass medially on the right thigh ( Figs. 5 and 6 ).

## Discussion

With the rising popularity of cosmetic fillers, the rate of complications is expected to increase. Consequently, it is crucial to compile information on these complications and their potential treatment options.

In a study by Namgoong et al, 146 patients underwent surgery to remove Aquafilling from the breast or buttock due to complications including induration, pain, firmness, asymmetry, dimpling, migration, infection, and sepsis. 3 This patient suffered long-term complications including pain, migration, infection leading to septic shock, and nonparathyroid hypercalcemia. The phenomenon behind filler migration is not well understood. However, filler migration from the buttock area is thought to occur due to a combination of gravity, muscle activity, physical compression, and the hydrophilic properties of Aquafilling, which enable diffusion through the fibrous septae found between subcutaneous tissue layers.

While Aquafilling has been described by Uysal et al to elicit foreign body reactions and granuloma-like structures, 7 we have not found any reports describing an association between nonparathyroid hypercalcemia and Aquafilling. Nonparathyroid hypercalcemia caused by granuloma formation triggered by foreign bodies is known from paraffin and silicone injections for body sculpturing, and behaves similarly to granulomatous diseases, such as sarcoidosis, where locally increased calcium metabolism occurs due to dysregulated production of 1,25-(OH) _2_ D _3_ (calcitriol) by activated macrophages. 8 9

Gierej et al analyzed the data of 31 women suffering from complications after breast enlargement with Aquafilling who were treated with either conservative or radical surgery, and recommend infiltrated tissue excision in addition to irrigation and drainage. 10 We were able to treat this patient with a minimally invasive approach and preserve the skin and majority of subcutaneous fat in the affected body areas. It can be assumed that the progression of the infection could have been avoided through radical surgery involving debridement of the skin and subcutaneous fat to the fascia level in the affected areas and reconstruction using split skin grafts. Conversely, we believe that radical surgery would have impaired the patient's physical abilities and quality of social and sexual life. The patient returned to her everyday life with no lasting sequelae but with a solid mass on the right thigh.

## Conclusion

This case contributes to the increasing number of reports highlighting the risk of complications associated with Aquafilling, with previously unknown issues still being reported. This underscores that further research regarding the use of Aquafilling is needed.

## References

[1] Harrison D Selvaggi G Gluteal augmentation surgery: indications and surgical management J Plast Reconstr Aesthet Surg 2007 60 08 922 928 17383947 10.1016/j.bjps.2006.01.057

[2] Atiyeh B Ghieh F Oneisi A Safety and efficiency of minimally invasive buttock augmentation: a review Aesthetic Plast Surg 2023 47 01 245 259 10.1007/s00266-022-03049-5 35999464

[3] Namgoong S Kim H K Hwang Y Clinical experience with treatment of Aquafilling filler-associated complications: a retrospective study of 146 cases Aesthetic Plast Surg 2020 44 06 1997 2007 32936330 10.1007/s00266-020-01889-7

[4] Wünscher S V Cambiaso-Daniel J Gualdi A Rappl T Long-term complications of gluteal augmentation using Aquafilling filler: a case report Indian J Plast Surg 2023 56 03 267 269 37435334 10.1055/s-0043-1767729 PMC10332892

[5] Shin J Suh J Yang S Correcting shape and size using temporary filler after breast augmentation with silicone implants Arch Aesthetic Plast Surg 2015 21 03 124 126

[6] Son M J Ko K H Jung H K Koh J E Park A Y Complications and radiologic features of breast augmentation via injection of Aquafilling gel J Ultrasound Med 2018 37 07 1835 1839 29280175 10.1002/jum.14527

[7] Uysal P I Ozkan B Savran S Borcek P Uysal A C Foreign body reaction to polyacrylamide filler (Aquafilling®) injected nine years previously: a complication of SARS-CoV-2 infection or merely a coincidence? J Cosmet Dermatol 2023 22 05 1694 1696 36718797 10.1111/jocd.15615

[8] Sølling A SK Tougaard B G Harsløf T Non-parathyroid hypercalcemia associated with paraffin oil injection in 12 younger male bodybuilders: a case series Eur J Endocrinol 2018 178 06 K29 K37 29599408 10.1530/EJE-18-0051

[9] Yedla N Perez E Lagari V Ayala A Silicone granulomatous inflammation resulting in hypercalcemia: a review of the literature AACE Clin Case Rep 2018 5 02 e119 e123 31967015 10.4158/ACCR-2018-0277 PMC6873861

[10] Gierej P Woźniak-Roszkowska E Radziszewski M Miszczyk J Krześniak N Noszczyk B Treatment of complications after minimally invasive breast augmentation with Aquafilling gel Aesthetic Plast Surg 2023 47 06 2322 2329 37721627 10.1007/s00266-023-03648-w

